# Multiorgan neutrophilic inflammation in a Border Collie with “trapped” neutrophil syndrome

**DOI:** 10.1111/jvim.16567

**Published:** 2022-10-14

**Authors:** Alyssa Zoto, Chelsea Stecklein, Michael A. Scott, Thomas R. Bauer, Cynthia Lucidi, Harry Cridge

**Affiliations:** ^1^ Department of Small Animal Clinical Sciences, College of Veterinary Medicine Michigan State University East Lansing Michigan USA; ^2^ Department of Pathobiology and Diagnostic Investigation, College of Veterinary Medicine Michigan State University East Lansing Michigan USA; ^3^ Immune Deficiency—Cellular Therapy Program, National Cancer Institute National Institutes of Health Bethesda Maryland USA; ^4^ Veterinary Diagnostic Laboratory, College of Veterinary Medicine Michigan State University Lansing Michigan USA

**Keywords:** bone marrow, Cohen syndrome, ineffective myelopoiesis, neutropenia, VPS13B

## Abstract

Trapped neutrophil syndrome is a rare congenital disease recognized in Border Collies and is characterized by persistent neutropenia with myeloid hyperplasia. The mechanism of neutropenia has not been described. We document the case of a young Border Collie diagnosed with trapped neutrophil syndrome based on clinical features, blood and bone marrow evaluation, and presence of the associated homozygous mutation. Results from flow cytometric and storage studies suggested lower neutrophil survival time. The dog had substantial neutrophilic inflammation in multiple organs, indicating that neutrophils could leave the marrow and enter tissues, making the term “trapped” neutrophil syndrome a misnomer.

**Abbreviations**
BSA: 1% bovine serum albumin
IHC: immunohistochemistry
MSU‐VMC: Michigan State University Veterinary Medical Center
PBS: phosphate buffered saline
TNS: trapped neutrophil syndrome
VPS13B: vacuolar protein sorting‐associated protein 13B

## 1 INTRODUCTION

Trapped neutrophil syndrome (TNS) is a hereditary autosomal recessive disorder that was first identified in Border Collies in the 1990s.^1^ There are 7 case reports involving 12 dogs with TNS.^1^, ^2^, ^3^, ^4^, ^5^, ^6^, ^7^ Most reported cases are from Europe,^3^, ^4^, ^6^ Asia,^5^ New Zealand,^1^ or Australia,^7^ with only 1 case reported in the United States.^2^ The disease has been characterized by persistent neutropenia with concurrent myeloid hyperplasia.^1^, ^3^, ^4^, ^7^ Persistent neutropenia in these dogs results in an impaired innate immune response with development of persistent or recurrent infections that involve the gastrointestinal tract, multiple joints, or a combination of both.^1^, ^2^, ^3^, ^4^, ^5^ The disorder also manifests as musculoskeletal abnormalities including lameness, stunted growth, and an elongated, narrow muzzle.^4^, ^5^

The genetic mutation in TNS is the same as that characterized for Cohen syndrome in people^8^; both disorders are caused by a mutation of the gene for vacuolar protein sorting‐associated protein 13B (VPS13B).^8^, ^9^ The protein VPS13B localizes to the outer membrane of the Golgi and is essential for maintenance of the structural and functional integrity of the Golgi.^10^ Cohen syndrome and TNS display similarities in clinical manifestations including slender extremities, craniofacial malformations, and neutropenia.

In this case report, we describe clinical, laboratory, and necropsy findings in a Border Collie diagnosed with TNS, provide evidence for shorter neutrophil survival in this dog, and document that many neutrophils can leave bone marrow and reach inflamed tissues, thus challenging the suitability of the name “trapped” neutrophil syndrome.

## 2 CASE SUMMARY

A 3‐month‐old intact male Border Collie was presented to Michigan State University Veterinary Medical Center (MSU‐VMC) for a 2‐day history of pyrexia, lethargy, and lameness progressing to nonambulation. The dog was reported to have episodic signs of gastrointestinal disease and “failure‐to‐thrive” since 6 weeks‐of‐age. Three weeks before presentation, the dog was hospitalized at the referring veterinarian with vomiting, diarrhea, hematochezia, and dehydration. The dog was vaccinated with a SQ core vaccine (Vanguard DAPP/L4, Zoetis, Parsippany, New Jersey) and an intranasal bordatella vaccine (Vangard B intranasal, Zoetis, Parsippany, New Jersey) 18 days before presentation. A cage‐side parvovirus antigen test (Supplemental Information) and a fecal flotation test were negative. Initial CBC and serum chemistry results reportedly revealed nonregenerative anemia and neutropenia with no other relevant findings. The dog received inpatient supportive care for gastroenteritis and was discharged the next day with instructions to administer amoxicillin.

**FIGURE 1:**
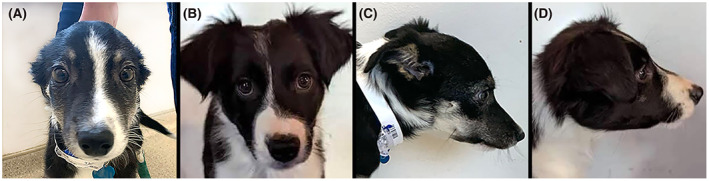
Abnormally narrow and elongated facial features in a 3‐month‐old Border Collie with trapped neutrophil syndrome (A and C) compared to a clinically healthy age‐matched dog of the same breed (B and D)

Three weeks later, the dog was presented to the MSU‐VMC because of fever, lethargy, and an inability to rise. On physical examination, the dog had dull mentation and was pyrexic (106.2°F) and tachycardic (160 bpm). The mandibular lymph nodes were enlarged and firm. Multiple joints were enlarged and warm, and the dog was nonambulatory. The dog had an abnormally narrow and elongated muzzle and abnormally slender extremities (Figure 1). A CBC (ADVIA 2120, Siemens, Munich, Germany) with a microscopic leukocyte differential revealed a low‐normal segmented neutrophil concentration (3.1 × 10^3^/μL; reference interval [RI], 2.7‐7.8 × 10^3^/μL) and monocytosis (2.4 × 10^3^/μL; RI, 0.1‐0.8 × 10^3^/μL). Based on extrapolation from published reference intervals for young dogs,^11^ erythroid abnormalities included a mild normocytic (MCV, 64 fL), normochromic (MCHC, 31 g/dL), nonregenerative anemia (spun Hct 26%; reticulocyte concentration, 39 700/μL; RI, 12 000‐76 000/μL), although the anemia appeared moderate, microcytic, and hypochromic using adult reference intervals. The platelet concentration was within the RI. Relevant serum biochemistry results were a total protein concentration of 5.3 g/dL with hypoalbuminemia (1.9 g/dL) and hypercholesterolemia (355 mg/dL) based on published data for healthy young dogs.^12^ Serum iron concentration was 16 μg/dL (RI, 109‐250 μg/dL). Tests for tick‐borne disease (Supplemental Information) and canine distemper virus (Supplemental Information) were negative. Thoracic limb radiographs revealed elbow and carpal effusion.

**FIGURE 2:**
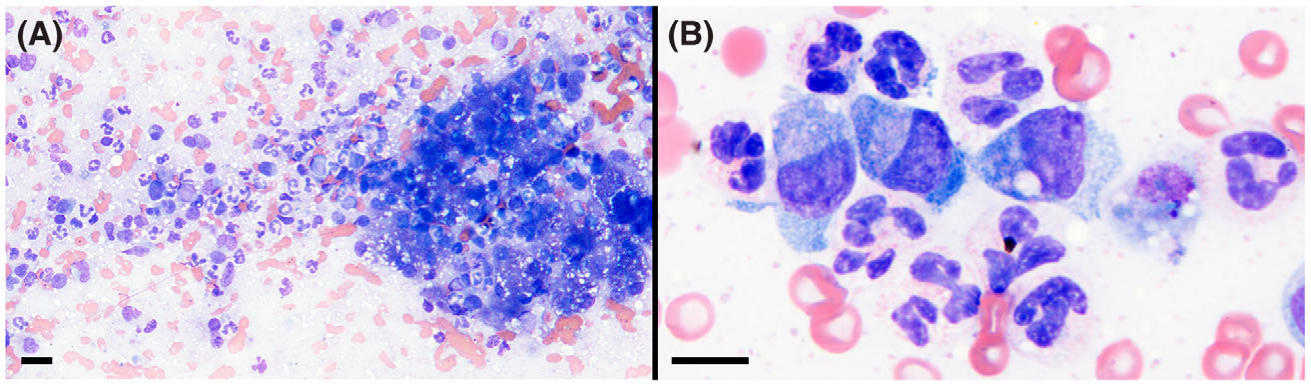
Neutrophilic and macrophagic inflammation in a cytologic specimen of the liver of the affected dog. Wright stain, magnification bars = 20 μm (A), 10 μm (B)

Abdominal ultrasonography revealed an enlarged hyperechoic liver, an enlarged heterogeneous spleen, and abdominal lymphadenomegaly; the right colic lymph node contained anechoic material. Aspirates of the liver, spleen, abdominal lymph nodes, mandibular lymph node, and synovial fluid were evaluated cytologically. Hepatic (Figure 2) and splenic smears contained expected tissue with moderately increased numbers of neutrophils and macrophages without significant myelopoiesis, supporting neutrophilic and macrophagic inflammation. In both samples, few macrophages contained phagocytized neutrophils. Rare giant segmented neutrophils were present. Fluid aspirated from the colic lymph node consisted predominantly of necrotic material with few recognizable neutrophils and moderate numbers of bacterial cocci seen extracellularly or within few neutrophils; culture of this fluid revealed moderate growth of *Enterococcus faecium*. Smears from the mandibular lymph node were highly cellular with predominantly neutrophilic and lesser macrophagic inflammation with many dead cells and minimal lymphoid tissue; microorganisms were not seen. Synovial fluid from 4 joints had increased percentages of neutrophils without clearly increased total nucleated cell concentrations; this was interpreted as mild neutrophilic inflammation. Ampicillin sulbactam was administered. Subsequent CBCs on day 2 and 4 of hospitalization revealed persistent low‐normal segmented neutrophil concentrations (3.4 × 10^3^/μL and 3.5 × 10^3^/μL, respectively) with a mild left shift (0.56 × 10^3^/μL; RI, 0.0‐0.1 × 10^3^/μL) on day 2 and mild toxic change on both days. The dogs pyrexia resolved after the addition of enrofloxacin to ampicillin therapy on day 2. A fecal flotation test revealed *Isospora caninum* oocytes and sulfadimethoxine was administered at 50 mg/kg PO. Iron dextran was administered intramuscularly.

**FIGURE 3:**
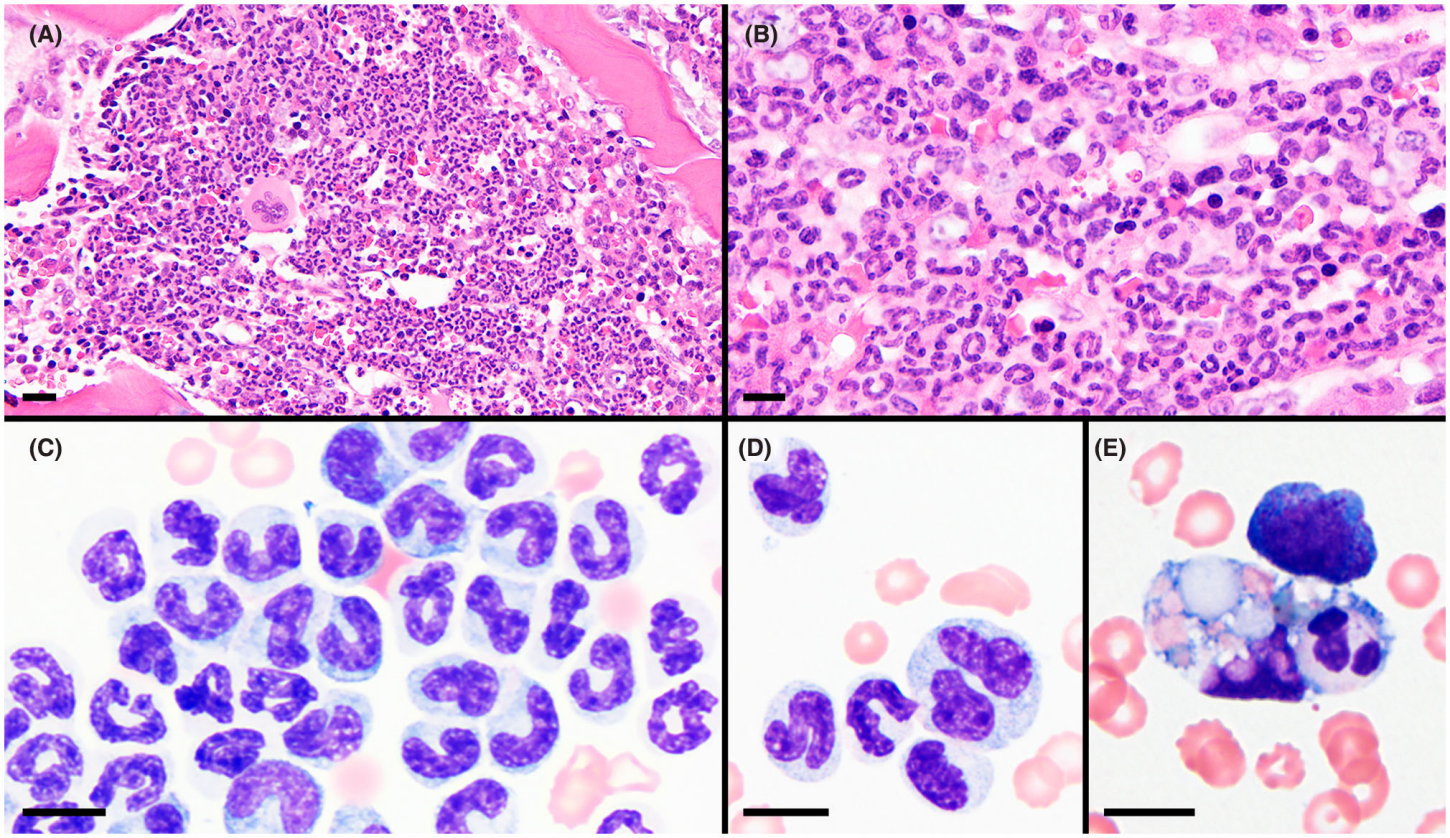
Bone marrow core biopsy (A and B) and aspirate (C‐E) specimens showing high cellularity and myeloid hyperplasia. Maturation of the myeloid lineage was complete through segmented neutrophils with prominent band neutrophils in many regions (B and C). Few giant neutrophils (D) and rare neutrophil phagocytosis (E) were present. H&E stain of B5‐fixed tissue (A and B) and Wright stain (C‐E); magnification bars = 20 μm (A), 10 μm (B‐E)

Given the signalment, elongated narrow muzzle and slender extremities, history of recurrent illness, and persistent low‐normal neutrophil concentration, the clinical suspicion for TNS was high and a blood sample was submitted for genetic testing on day 4 (Supplemental Information). To obtain quicker support for TNS and assess for other disease, bone marrow core and aspirate samples were collected from the right ileal wing on day 5. These samples collectively revealed marrow with 95% to 100% hematopoietic cellularity, a markedly increased myeloid to erythroid ratio (24 : 1), and therefore marked myeloid hyperplasia (Figure 3). Maturation of neutrophil precursors was complete through segmented neutrophils, with expansion of all maturation stages and with similar numbers of band and segmented neutrophils. Aspirate smears revealed rare but increased neutrophil phagocytosis and few giant band and segmented neutrophils, but not enough dysplasia to consider a myelodysplastic disorder. The erythroid lineage consisted of low‐to‐moderate numbers of small erythroid islands without evidence of hyperplasia. Hemosiderin was lacking, as expected for a 3‐month‐old dog, and megakaryopoiesis appeared adequate. Bone formation was incomplete, as expected for a young dog. Given the breed, age, clinical abnormalities, and marrow findings, TNS was considered the primary differential. After clinical improvement over a 5‐day hospitalization, the dog was discharged with continued antimicrobial therapy (amoxicillin clavulanic‐acid and enrofloxacin PO), and sulfadimethoxine. Genetic testing subsequently confirmed the diagnosis of TNS by detecting homozygosity for the associated *VPS13B* mutation.

Sixteen days after discharge, the dog was readmitted for lethargy and pyrexia and supportive care was reinitiated. Given lack of improvement over 48 hours, dexamethasone SP (0.2 mg/kg IV) was administered based on previous reports suggesting benefits in affected dogs with TNS.^4^ Marked clinical improvement with resolution of pyrexia and nonambulation was noted the next day. Prednisone (initially 1.8 mg/kg/day, then tapered PO) and the previously noted antimicrobials were continued. Over the next 6 months, the dog continued to have waxing‐and‐waning illness characterized by intermittent pyrexia, hyporexia, and mandibular lymphadenomegaly. Cytologic assessment of the enlarged lymph nodes 3 months after admission revealed marked neutrophilic and moderate macrophagic inflammation with no apparent cause; aerobic and anaerobic bacterial cultures were negative. Throughout this time, neutrophil concentrations remained within RI (10 of 15 CBCs) or mildly decreased (4 of 15), except for 1 episode of mild neutrophilia with a moderate left shift and toxic neutrophils. The episodes of neutropenia were intermittent and occurred 7‐12 weeks apart. Because of concerns for quality of life, the owners elected euthanasia when the dog was 10 months old.

**FIGURE 4:**
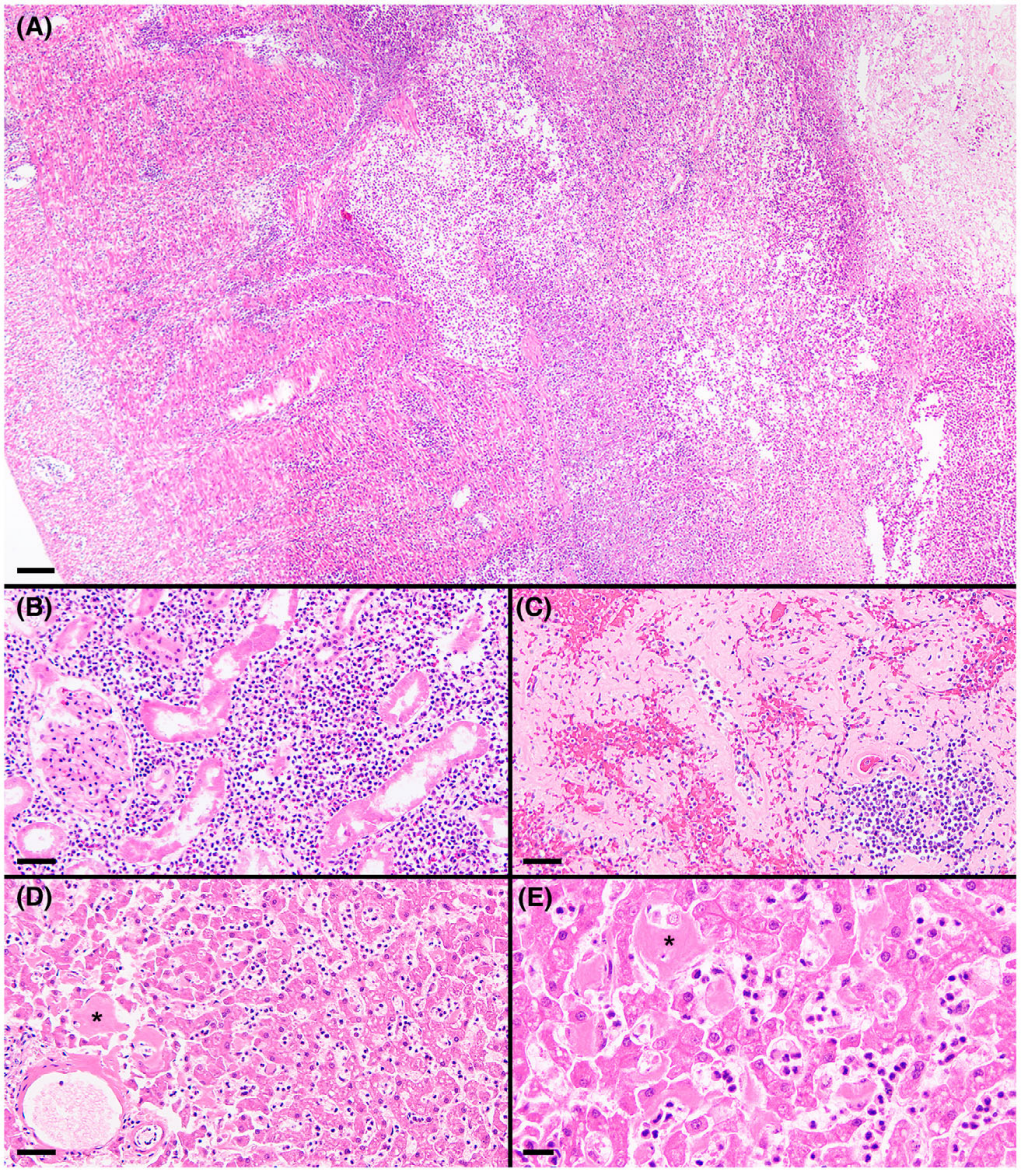
Histologic specimens of ileum (A), renal cortex (B), spleen (C), and liver (D and E). Effacing transmural necrotizing and suppurative lesion within the ileum, with the serosa to the left and the ulcerated mucosa to the right (A). Marked suppurative inflammation within the renal cortex and associated tubular degeneration (B). Marked amyloid deposition throughout this region of the spleen (C). Moderate hepatic amyloid deposition (asterisks denote representative accumulations) and many sinusoidal leukocytes, mostly neutrophils, despite a low‐normal blood neutrophil concentration (D and E). H&E stain; magnification bars = 100 μm (A), 50 μm (B‐D), 20 μm (E)

Necropsy revealed marked multifocal ulcerative enteritis with a transmural necrotizing and suppurative lesion in the ileum that was associated with peritonitis and systemic evidence of septicemia and chronic inflammation (Figure 4). This included bilateral multifocal renal cortical abscesses, marked suppurative mesenteric lymphadenitis, and moderate to severe amyloid deposition in the spleen, liver, adrenal gland cortices, pancreatic islets, thyroid interstitium, and multiple lymph nodes. Amyloid was confirmed by Congo red staining and birefringence. Hepatic sinusoids contained many neutrophils despite a low‐normal blood neutrophil concentration. Septicemia was suspected to be secondary to bacterial transmigration through the wall of the ileum. The medullary space of long bones appeared decreased and partially replaced by trabecular bone, but myeloid hyperplasia was still evident. Increased extramedullary hematopoiesis was not detected. Homozygosity for the *VPS13B* mutation (Supplemental Information) was confirmed postmortem in combined tissues.

## 3 MECHANISTIC INVESTIGATIONS

**FIGURE 5:**
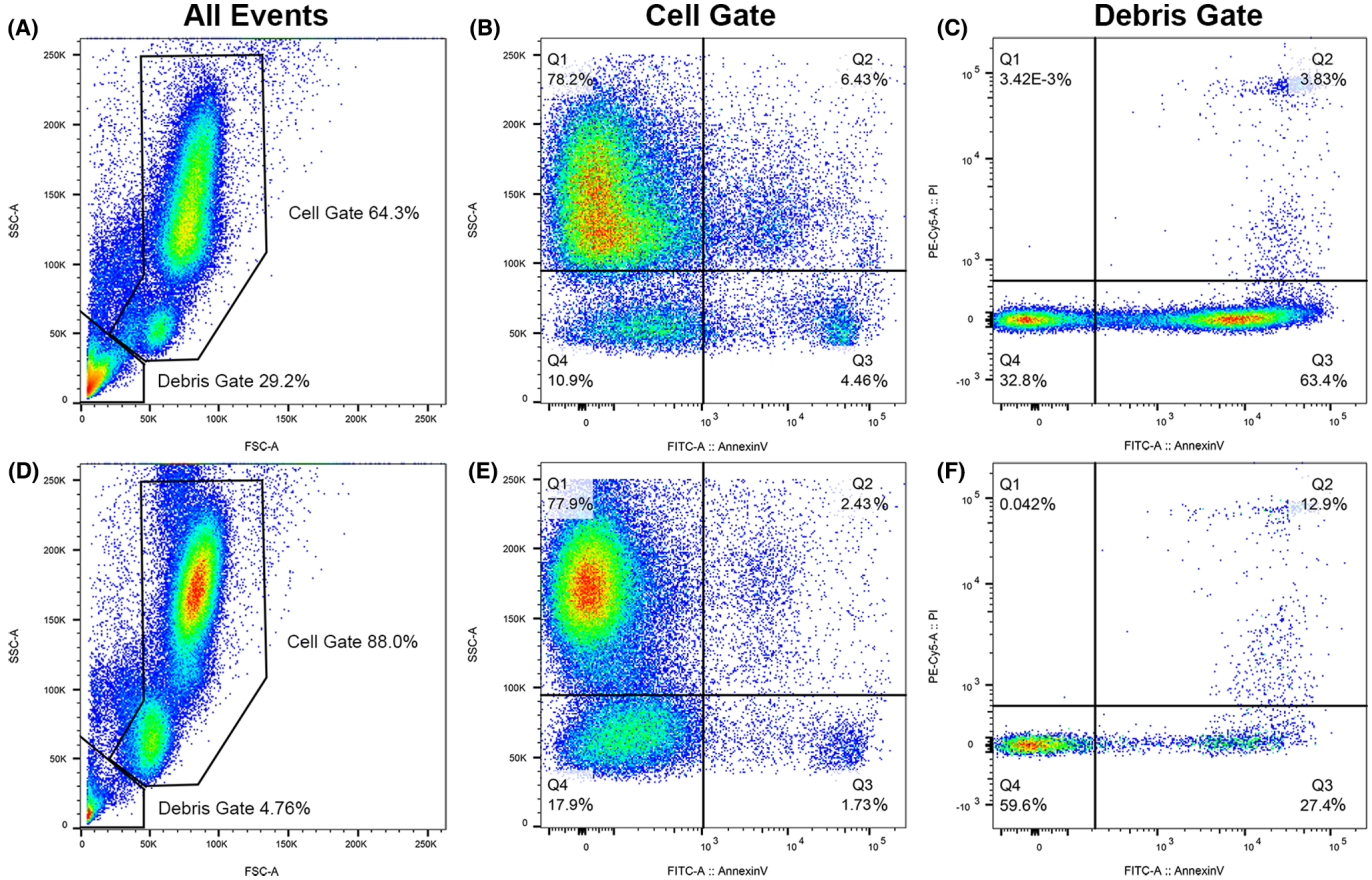
Flow cytometric scattergrams of blood leukocytes from the TNS dog (A‐C) and 1 representative control dog (D‐F). Initial gating (A and D) included 100 000 events for each dog and was based on forward scatter (horizontal axes), a representation of cell size, and side scatter (vertical axes), a representation of cell complexity into cell and debris gates; the affected dog had increased debris. The cell gate (B and E) shows expression of annexin V, a marker of cell death, on the horizontal axes, and side scatter on the vertical axes. The affected dog had an increased percentage of cells expressing annexin V compared to control dogs (upper and lower right quadrants). The top and bottom cell clouds in A, B, D, and E represent neutrophils and lymphocytes, respectively; monocytes appear as a subpopulation overlapping the bottom of the neutrophil cloud (labeling for each cell type not shown). The debris gate (C and F) shows expression of annexin V on the horizontal axes and propidium iodide, an additional marker of cell death, on the vertical axes. The affected dog had a greater percentage of annexin V^pos^/propidium iodide^neg^ events (lower right quadrant) compared to each of 4 control dogs

Methods and control samples are described in the Supplemental Information. Expression of the selectin CD62L, integrins CD11b, CD18, and CD49d, and the bone marrow homing receptor CXCR4 was similar to control samples (data not shown). However, flow cytometric analysis of day‐old EDTA whole blood revealed an increased proportion of events classified as debris (29.2%) in the TNS dog's sample compared to control dog samples (2.8%‐5.2%; Figure 5A,D), suggesting increased cell deterioration. Within the gate of intact cells (Figure 5B,E), the TNS dog had the highest percentage of annexin V^pos^ cells (10.9%) compared to control dogs (2.6%‐5.0%), including likely neutrophils (lower left of Q2), supportive of increased phosphatidylserine exposure and possible cell death. Within the gate of debris (Figure 5C,F), the TNS dog had the highest proportion of annexin V^pos^/propidium iodide^neg^ events (63.4%) compared to control dogs (9.3%‐29.7%), further supporting increased phosphatidylserine exposure and possible increased cell death in the TNS dog's sample.

**FIGURE 6:**
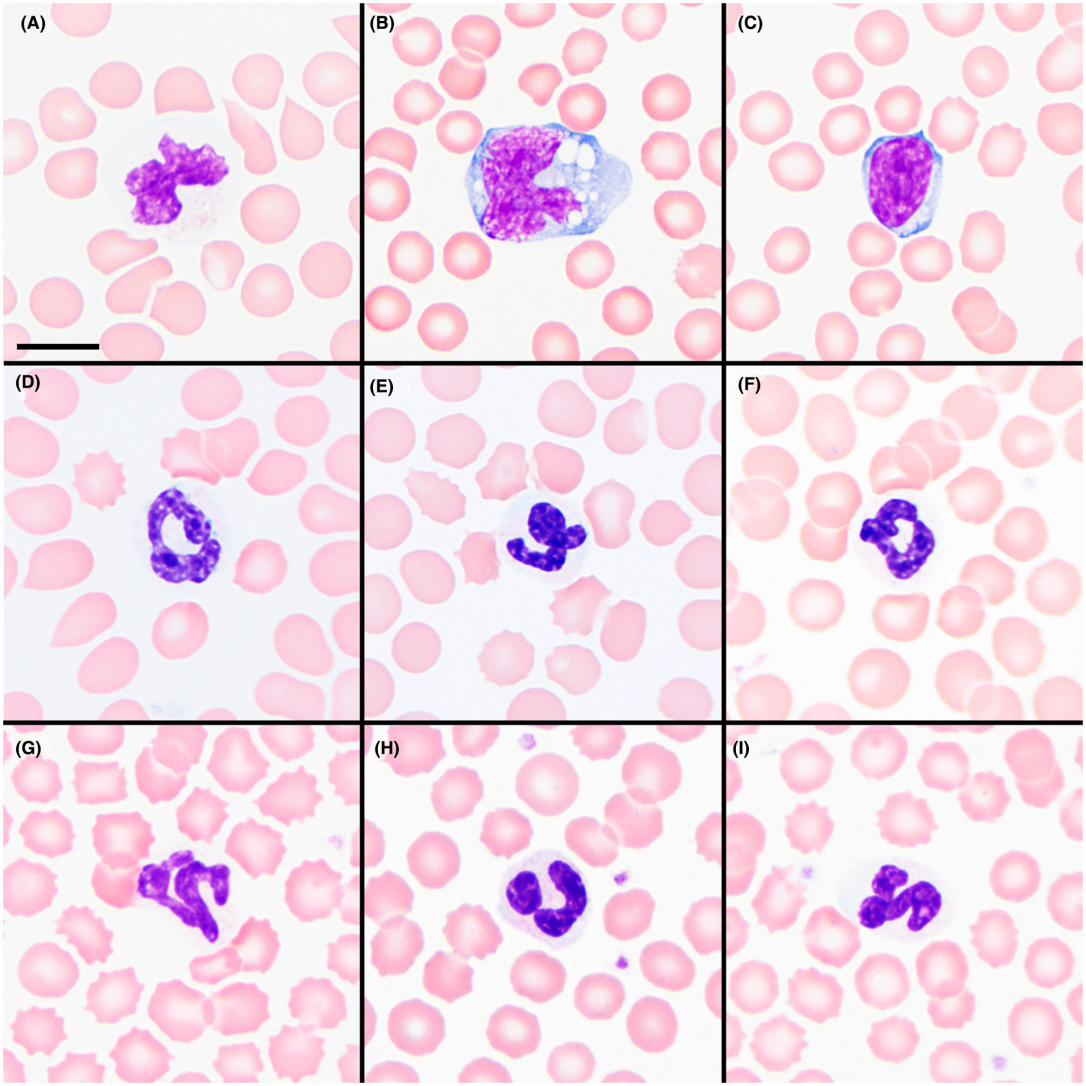
EDTA‐anticoagulated whole blood after 12 hours of refrigeration depicting a representative neutrophil (A), monocyte (B), and lymphocyte (C) from the affected dog compared to segmented neutrophils (D‐I) from 6 control dogs after the same storage time. Wright stain; magnification bar = 10 μm

Based on the preceding results, a blood storage experiment was done to assess in vitro cell preservation over time. After 12‐hour refrigeration of EDTA‐anticoagulated blood, most neutrophils in a stained blood smear from the TNS dog had moderate‐marked deterioration and were lysed or partially lysed with swollen nuclei (Figure 6A). Monocytes were only mildly deteriorated, and lymphocytes were mostly well preserved (Figure 6B,C). Compared to a 12‐hour sample, lysed cells increased from 5% (350/μL) to 22% (1450/μL) at 36 hours; neutrophil deterioration had increased, but preservation of monocytes and lymphocytes was relatively unchanged. In contrast, 12‐hour refrigeration yielded leukocytes with no to mild (5 dogs) or mild‐moderate (1 dog) deterioration in stained smears made from 6 control‐dog samples, including 2 that were receiving glucocorticoid therapy at a similar dose as the TNS dog and 4 that were not receiving this drug (Figure 6D‐I).

## 4 DISCUSSION

This case report provides detailed antemortem and postmortem cytologic and histopathologic findings indicating that neutrophils in dogs with TNS can leave hematopoietic tissue and enter other tissues to cause marked suppurative lesions, even though the disorder's name suggests they are trapped in the bone marrow. Additionally, the case shows that TNS dogs might not have persistent neutropenia. Our finding of decreased in vitro neutrophil survival suggests that decreased in vivo neutrophil survival might contribute to impaired neutrophil responses in these dogs.

Although most reported TNS dogs have had persistent neutropenia,^1^, ^2^, ^3^, ^4^, ^7^ the dog reported here had persistently low‐normal neutrophil concentrations with only sporadic neutropenia. These neutrophil concentrations were present despite myeloid hyperplasia and suppurative inflammatory lesions. Multiorgan neutrophilic inflammation and increased sinusoidal neutrophils in liver indicate that neutrophils were able to leave the bone marrow and reach other tissues, and the persistent low to low‐normal neutrophil concentrations indicate that margination and egress from blood outpaced neutrophil movement from marrow to blood. The latter might reflect a degree of ineffective myelopoiesis, supported in this dog by marked myeloid hyperplasia concurrent with persistent low or low‐normal neutrophil concentrations, phagocytosis of neutrophils in the marrow, and lack of rebound neutrophilia after administration of antibiotics. Another potential contributor to the low or low‐normal neutrophil concentrations is abnormally brief blood circulation time related to premature clearance and shorter neutrophil survival time. Phagocytosis of neutrophils in the marrow and other organs might reflect such clearance, whether of dying neutrophils or of neutrophils otherwise marked for phagocytosis. In any case, the presence of neutrophilic inflammation within multiple organs indicates that neutrophil production was not entirely ineffective, and neutrophils were not completely trapped in the bone marrow. Inflammatory lesions have been documented in 3 prior reports,^1^, ^2^, ^4^ but cytologic or histologic assessments were not consistently done or reported for other cases.

In the 7 other reported cases of TNS for which cytologic or histologic assessment of bone marrow specimens was reported, myeloid hyperplasia was present in all dogs.^1^, ^3^, ^4^, ^7^ Myeloid maturation was variously described as orderly and complete^1^, ^3^ or left shifted.^4^, ^7^ Neutrophil phagocytosis was also documented in 1 previous dog,^1^ but giant neutrophils are not previously reported. Giant neutrophils might reflect endomitosis caused by aberrant cell divisions as can occur with accelerated neutropoiesis.

Shorter neutrophil survival because of accelerated apoptosis is a proposed mechanism of neutropenia in patients with Cohen Syndrome,^13^ warranting investigations regarding cell death for the dog in this report. Our flow cytometric analysis of the TNS dog's leukocytes revealed evidence of accelerated in vitro leukocyte death, and a subsequent storage study provided evidence that the TNS dog's neutrophils degraded rapidly in vitro compared to neutrophils of other dogs. The cells with increased annexin V positivity were not definitively identified with leukocyte‐specific markers, but they appeared to include neutrophils, based on their side‐scatter regions. Additionally, our storage study showed accelerated deterioration of neutrophils, but not other cell types. Overall, this suggests decreased in vitro survival of neutrophils in the TNS dog and raises the consideration of decreased in vivo neutrophil survival, whether in the bone marrow, in circulation, or in destination tissues.

Expression of cell surface selectin, integrins, and CXCR4 did not appear abnormal in the TNS dog. Others have found no CXCR4 mutations in dogs with TNS.^14^ Decreased neutrophil expression of CD62L and CD11b was shown for 1 person with Cohen syndrome, and it was attributed to increased neutrophil activation in vivo.^15^ A limitation of our flow cytometric study, including annexin V, is that the affected dog was receiving prednisone (0.88 mg/kg/d), while controls were not. In cattle and people, glucocorticoids can reduce neutrophil expression of CD62L,^16^, ^17^ and in cattle, glucocorticoids can decrease neutrophil apoptosis, rather than increase it^18^; the effects of glucocorticoid use on expression of canine neutrophil surface proteins is unclear.

## CONFLICT OF INTEREST DECLARATION

Authors declare no conflict of interest.

## OFF‐LABEL ANTIMICROBIAL DECLARATION

Specific details regarding medical treatments for the dog are not discussed in this case report, consistent with the author guidelines. However, several off‐label medications were utilized in the management of this case.

## INSTITUTIONAL ANIMAL CARE AND USE COMMITTEE (IACUC) OR OTHER APPROVAL DECLARATION

Approved by Michigan State University IACUC, PROTO202000334.

## HUMAN ETHICS APPROVAL DECLARATION

Authors declare human ethics approval was not needed for this study.

## Supporting information


**Appendix S1**. Supporting Information
