# Supplementary material for: Multiorgan neutrophilic inflammation in a Border Collie with “trapped” neutrophil syndrome
Source: J Vet Intern Med. 2022 Oct 14;36(6):2170–6. doi: 10.1111/jvim.16567 (PMC9708390; doi:10.1111/jvim.16567)
Supplement: Supplementary file 1 — Appendix S1. Supporting Information [file JVIM-36-2170-s001.pdf]

Supplemental Information:

Commercial Diagnostic Testing:

1. Patient-side parvovirus assay: SNAP Parvovirus Antigen Test Kit, IDEXX Laboratories, One IDEXX Drive, Westbrook, Maine 04092, USA

2. Patient-side infectious disease screening: SNAP 4Dx Plus Test Kit, IDEXX Laboratories, One IDEXX Drive, Westbrook, Maine 04092, USA

3. Distemper PCR: Canine Distemper Virus PCR, Michigan State University Veterinary Diagnostic Laboratory, 4125 Beaumont Road, Lansing, MI 48910, USA

4. Trapped Neutrophil Genetic Testing:

4.1. Blood: Trapped Neutrophil Syndrome Genetic Screening, Animal Genetics, 3382 Capital Circle NE, Tallahassee, FL 32308, USA

4.2. Combined necropsy tissues: Trapped Neutrophil Syndrome PCR, Veterinary Genetics Laboratory, University of California Davis, Old Davis Road, Davis, CA 95616, USA

Flow Cytometry:

Whole blood samples collected in EDTA from the TNS dog (4 months old at the time of testing) and four clinically healthy control dogs were shipped overnight within hours after collection with a cool pack in an insulated container to the NIH. Control dogs included a 9-month-old castrated male Siberian husky, a one-year-old intact female small mixed breed dog, a 7-year-old spayed female greyhound, and a 15-year-old castrated male Manchester terrier. At the time blood was collected for flow cytometric analysis, the TNS dog had a moderate monocytosis ($1.6\times10^3/\mu L$; RI, $0.1\text{-}0.8\times10^3/\mu L$), segmented and band neutrophil concentrations within reference intervals ($5.4\times10^3/\mu L$; RI, $2.7\text{-}7.8\times10^3/\mu L$ and $0.1\times10^3/\mu L$; RI, $0\text{-}0.1\times10^3/\mu L$, respectively), and was

receiving oral prednisone at 0.88 mg/kg/d. Control dogs were not receiving glucocorticoids and had lower percentages of monocytes (5-9%) than the TNS dog (19%). Leukogram abnormalities of control dogs were 1) minimal monocytosis ($0.55\times10^3/\mu L$; RI, $0.2\text{-}0.5\times10^3/\mu L$) in the 15-year-old; 2) mild neutrophilia ($10.61\times10^3/\mu L$; RI, $2.6\text{-}7.5\times10^3/\mu L$), lymphocytosis ($4.48\times10^3/\mu L$; RI, $0.8\text{-}3.9\times10^3/\mu L$), and monocytosis ($0.93\times10^3/\mu L$; RI, $0.2\text{-}0.5\times10^3/\mu L$) in the 9-month-old; and 3) mild lymphopenia ($0.65\times10^3/\mu L$; RI, $0.8\text{-}3.9\times10^3/\mu L$) in the 7-year-old. The leukogram of the one-year-old lacked evidence of disease. Leukocyte differentials and provided reference intervals are based on a microscopic assessment for the TNS dog and automated assessment plus microscopic confirmation for the control dogs.

Blood leukocytes were isolated using ammonium chloride lysis solution (Quality Biological Inc., Gaithersburg, Maryland) followed by washing with phosphate buffered saline (PBS) + 1% bovine serum albumin (BSA). For immunolabeling, leukocytes were resuspended at 10×10^6 cells/mL and 100 µL of cells were pre-incubated with 200 µL PBS/1% BSA containing 8% heat-inactivated Human AB serum and 50 µg/mL rat IgG.

Leukocyte subset analysis was performed using monoclonal antibodies to lymphocyte, monocyte, and neutrophil populations, including T-cells (CD3, CA17.2A12, Bio-Rad Laboratories Inc., Hercules, California), B-cells (CD21, B-ly4, BD Biosciences, San Jose, California), monocytes (CD14, M5E2, BioLegend, San Diego, California), and neutrophils (CADO48A, Washington State University Monoclonal Antibody Center, Pullman, Washington). Surface adhesion molecules were examined using monoclonal antibodies to the integrins CD11b (CA16.3E10, Bio-Rad Laboratories Inc.), CD18 (CA1.4E9, Bio-Rad Laboratories Inc.), and CD49d (Bu49, Bio-Rad Laboratories Inc.); to the selectin CD62L (FMC46, Bio-Rad Laboratories Inc.); and to the bone marrow homing molecule CD184/CXCR4 (12G5, Invitrogen

eBioscience, Thermo Fisher Scientific, Waltham, Massachusetts). Antibodies CADO48A and CD11b were secondarily labeled using Zenon antibody labeling kits (Thermo Fisher Scientific). Dead cells were removed from analysis using staining with 7-aminoactinomycin D.

Leukocyte populations, without immunolabeling for discrimination of cell types, were analyzed for cell death using an annexin V/Dead Cell Apoptosis Kit (Invitrogen Molecular Probes, Thermo Fisher Scientific) containing Alexa Fluor 488 annexin V and propidium iodide to discriminate apoptotic cells/dead cells from live cells. Leukocytes were labeled according to the manufacturer's directions.

All leukocytes underwent flow cytometric analysis on a BD Fortessa, followed by software analysis using FlowJo (v 9.9.6, TreeStar Inc., Ashland, Oregon). One hundred thousand events were collected for each dog for the initial leukocyte/debris gating run. Additional gating was performed on events from the initial run classified as either leukocytes or debris (e.g., the TNS dog had 29.2% debris initially so 29,233 events were included in the second debris gating for annexin V and propidium iodine expression).

Storage Experiment:

Whole blood was collected in EDTA immediately prior to euthanasia, refrigerated at 4 °C, and a CBC was performed as soon as possible, which was approximately 12 hours after collection. The sample was then stored at room temperature (20 °C) and serial CBCs were performed 1.5 and 2 days after initial collection, the former timepoint to mimic the delay between collection and analysis for the samples shipped for flow cytometric study. Blood smears were evaluated by two authors (Chelsea Stecklein, Cynthia Lucidi) who assessed erythrocytes, platelets, and leukocytes, including microscopic leukocyte differential counts. Smears of whole

blood collected in EDTA from six routine canine laboratory samples that had a CBC performed immediately upon collection or 12 hours after collection were evaluated at 12 hours after refrigerated storage (4 °C) as described for the TNS dog. These 6 control dogs were a 13-year-old castrated male mixed breed dog, an 8-year-old castrated male American Staffordshire terrier, a 6-year-old spayed female mixed breed dog, a 5-month-old spayed female mixed-breed dog receiving glucocorticoid therapy at a similar dose as the TNS dog (1.0 mg/kg every other day), a 15-month-old, spayed female mixed-breed dog receiving glucocorticoid therapy at a similar dose as the TNS dog (0.69 mg/kg/day), and a healthy 5-month-old, spayed female mixed-breed dog. Leukogram abnormalities of these dogs were 1) mild segmented neutrophilia ($9.0\times10^3/\mu L$; RI, $2.7\text{-}7.8\times10^3/\mu L$) with a marked left shift ($5.0\times10^3/\mu L$; RI, $0.0\text{-}0.1\times10^3/\mu L$), mild monocytosis ($1.6\times10^3/\mu L$; RI, $0.1\text{-}0.8\times10^3/\mu L$), and toxic change in the 13-year-old; 2) mild mature neutrophilia ($11.4\times10^3/\mu L$; RI, $2.6\text{-}7.5\times10^3/\mu L$) and monocytosis ($1.6\times10^3/\mu L$; RI, $0.2\text{-}0.5\times10^3/\mu L$) in the 8-year-old; 3) moderate mature neutrophilia ($27.9\times10^3/\mu L$; RI, $2.6\text{-}7.5\times10^3/\mu L$) and monocytosis ($2.8\times10^3/\mu L$; RI, $0.2\text{-}0.5\times10^3/\mu L$) in the 6-year-old; 4) mild monocytosis ($1.4\times10^3/\mu L$; RI, $0.1\text{-}0.8\times10^3/\mu L$) and mild eosinophilia ($1.5\times10^3/\mu L$; RI, $0.0\text{-}1.3\times10^3/\mu L$) in the 5-month-old receiving glucocorticoids; 5) mild lymphopenia ($0.5\times10^3/\mu L$; RI, $0.8\text{-}3.9\times10^3/\mu L$) in the 15-month-old receiving glucocorticoids; and 6) mild lymphocytosis $6.1\times10^3/\mu L$; RI, $0.8\text{-}3.9\times10^3/\mu L$) and mild monocytosis ($0.9\times10^3/\mu L$; RI, $0.2\text{-}0.5\times10^3/\mu L$) in the healthy 5-month-old. The 5-month-old control dog receiving glucocorticoid therapy had mild-moderate deterioration of all leukocytes 12 hours after refrigerated storage (Figure 6 G), while the remaining control dogs had no to mild deterioration of leukocytes.

Immunohistochemistry:

In Cohen syndrome, decreased expression of serpin B1 by neutrophils and increased neutrophil apoptosis might cause or contribute to neutropenia.[1] Bone marrow core sections from the TNS and one control dog were assessed immunohistochemically using an anti-serpin B1 antibody, but useful results were not obtained because various antigen retrieval, blocking, and incubation modifications failed to consistently label myeloid cells without labeling erythroid precursors. Reduced serpin B1 expression in canine TNS was not confirmed and remains a possibility.

Paraffin-embedded canine bone marrow core samples fixed in B-5 fixative were sectioned at 4 μm onto 4 charged slides and dried overnight at 56 °C. Slides were deparaffinized and hydrated in distilled water followed by mercury pigment removal utilizing iodine and sodium thiosulfate. Pretreatments were as follows: 1) no enzymatic pretreatment; 2) proteolytic enzyme induced epitope retrieval performed with 0.03% Pronase E (MilliporeSigma, St. Louis, Missouri) and Tris buffered saline (TBS) (ScyTek Laboratories, Inc., Logan, Utah) at 37 °C for 10 minutes followed by running tap and distilled water rinses; 3) high heat retrieval at pH 6.0 via steamer; and 4) high heat retrieval at pH 9.0 via steamer. Endogenous peroxidases were blocked by soaking slides in a 3.0% hydrogen peroxide/methanol solution at a 1:4 ratio for 30 minutes followed by running tap and distilled water rinses. Slides were incubated in TBS and Tween 20 (ScyTek) for 5 minutes to allow for pH adjustment. All slides were then transferred to the automated labeling platform intelliPATH FLX (Biocare Medical, Pacheco, California) at room temperature (20 °C) for the remainder of the labeling protocol with rinses in automation wash buffer between each reagent. Nonspecific protein was blocked in intelliPATH Background Punisher (Biocare Medical) for 5 minutes. Monoclonal mouse anti-serpin B1 (Thermo Fisher Scientific, Waltham, Massachusetts; clone OTI3B4) was diluted 1:100 in normal antibody

diluent (ScyTek) for 60 minutes followed by mouse-on-canine horseradish peroxidase micropolymer (Biocare Medical) for 30 minutes. A reaction was developed with a Romulin AEC chromogen kit (Biocare Medical) for 5 minutes followed by staining with CAT hematoxylin (Biocare Medical) at a dilution of 1:10 for 2 minutes. Upon completion of automated staining, slides were removed and allowed to dry completely prior to being dipped in xylene and coverslipped with a permanent mounting medium. Four paired control slides were simultaneously labeled using an IP universal negative control (Biocare Medical). All slides were evaluated by Chelsea Stecklein and Cynthia Lucidi.

Four additional 3 μm-thick sections with only the proteolytic enzyme pretreatment and the following four adjustments were evaluated: 1) monoclonal mouse anti-serpin B1 (Thermo Fisher Scientific) was diluted 1:100 in normal antibody diluent (ScyTek) for 30 minutes; 2) monoclonal mouse anti-serpin B1 (Thermo Fisher Scientific) was diluted 1:200 in normal antibody diluent (ScyTek) for 60 minutes; 3) mouse-on-canine horseradish peroxidase micropolymer (Biocare Medical) was incubated for 20 minutes; and 4) mouse-on-canine horseradish peroxidase micropolymer (Biocare Medical) for incubated for 15 minutes. All slides were stained with CAT hematoxylin (Biocare Medical) at a dilution of 1:10 for 3 minutes. Slides were evaluated, and the anti-serpin B1 1:100 dilution for 30 minutes was selected based on the best signal-to-noise ratio. An additional section from the affected dog and a control dog were evaluated after processing using this protocol. The control dog was a 17-month-old English springer spaniel receiving phenobarbital with hypercellular bone marrow characterized by mildly left-shifted myeloid hyperplasia and a predominance of band and segmented neutrophils.

Supplemental Reference:

1. Duplomb L, Rivière J, Jego G, et al. Serpin B1 defect and increased apoptosis of neutrophils in Cohen syndrome neutropenia. *J Mol Med* 2019;97:633–45.
